# Valle Agricola Chickpeas: Nutritional Profile and Metabolomics Traits of a Typical Landrace Legume from Southern Italy

**DOI:** 10.3390/foods10030583

**Published:** 2021-03-10

**Authors:** Nicola Landi, Simona Piccolella, Sara Ragucci, Shadab Faramarzi, Angela Clemente, Stefania Papa, Severina Pacifico, Antimo Di Maro

**Affiliations:** Department of Environmental, Biological and Pharmaceutical Sciences and Technologies (DiSTABiF), University of Campania Luigi Vanvitelli, Via Vivaldi 43, 81100 Caserta, Italy; nicola.landi@unicampania.it (N.L.); simona.piccolella@unicampania.it (S.P.); sara.ragucci@unicampania.it (S.R.); shadab.faramarzi@unicampania.it (S.F.); angela.clemente@unicampania.it (A.C.); stefania.papa@unicampania.it (S.P.); severina.pacifico@unicampania.it (S.P.)

**Keywords:** amino acids, *Cicer arietinum* L., legumes, metabolic profile, nutritional values, food quality

## Abstract

Chickpea (*Cicer arietinum* L.) from Valle Agricola is a legume cultivated in Southern Italy whose intake is strictly linked to rural traditions. In order to get new biochemical insight on this landrace and to promote its consumption and marketing, nutritional values (moisture content, total proteins, lipids, total and free amino acids) and metabolic traits are deeply investigated. Valle Agricola chickpea is nutritionally rich in proteins (19.70 g/100 g) and essential amino acids (7.12 g/100 g; ~40% of total). Carbohydrates, whose identity was unraveled by means of UHPLC-HR MS/MS analysis, were almost 60% of chemicals. In particular, a di-galactosylglycerol, a pinitol digalactoside, and a galactosylciceritol were found as constitutive, together with different raffinose-series oligosaccharides. Although lipids were the less constitutive compounds, glycerophospholipids were identified, while among free fatty acids linoleic acid (C18:2) was the most abundant, followed by oleic (C18:1) and palmitic (C16:0) acids. Isoflavones and hydroxybenzoic acid derivatives were also detected. Valle Agricola chickpeas showed very good levels of several mineral nutrients, especially magnesium (164 mg/100 g), potassium (748 mg/100 g), calcium (200 mg/100 g), zinc (4.20 mg/100 g) and manganese (0.45 mg/100 g). The boiling process favorably decreases anti-trypsin and anti-chymotrypsin activities, depleting this precious seed of its intrinsic antinutritional factors.

## 1. Introduction

The “Mediterranean diet”, which was recognized by the UNESCO as an “Intangible Cultural Heritage of Humanity” [1], is mainly based on a high intake of vegetables, pulses (beans, lentils, etc.), fruit, and cereals, in addition to fish and other foods, of which the Mediterranean basin is diversely rich. Pioneering scientific research by Ancel Keys disclosed the beneficial and protective effects of the Mediterranean diet [2], and following studies highlight that Mediterranean populations show a 50% lower mortality rate due to heart failure [3,4] or cancer [5]. The preventive and protective effects towards cancer, or its recurrence, were also investigated, so much so that the Mediterranean dietary pattern was hypothesized to be a lifestyle, and eating is synonymous with health and longevity [5,6].

Legumes, with their diversity in nutrients, represent a key food for the sustainable and healthy Mediterranean diet [7]. In fact, legumes (e.g., lupins, green beans and peas, peanuts, soybeans, dry beans, broad beans, dry peas, chickpeas, and lentils) are a rich source of raw proteins, dietary fibers, carbohydrates, vitamins and minerals [8]. In particular, raw proteins in legumes are a reservoir of the limiting amino acid lysine, whereas they are deficient in essential sulfur amino acids (methionine and cysteine) and tryptophan, requiring that they be consumed in a varied diet with cereals [9]. In addition, legumes contain secondary metabolites (e.g., flavonoids, tannins, saponins) that positively affect human health, contributing to the low impact of chronic and inflammatory diseases in humans with a high weekly legumes’ intake [10]. Furthermore, the high fiber content makes legumes an excellent food with a low glycemic index, and effective in controlling diabetes [8]. Indeed, besides the beneficial effects on human health, sustainable agriculture benefits from legume cultivation thanks to their symbiosis with nitrogen-fixing bacteria and low water requirements [11]. In light of this, having a large reservoir of legume landraces is of interest to make up for the decrease in diversity imposed by mechanized agriculture and to dispose of a rich crop gene pool (large genetic variability) necessary to improve both the health effects and a cultivation capable of promoting a sustainable agriculture [11,12].

Locally cultivated legumes are consumed as traditional food in Italy, where several communities preserve typical landraces, which are endemic varieties. These latter have been domesticated and locally adapted to the specific environment through the isolation from other populations of the same species [13]. In this context, during a framework aimed to valorize legumes from the Campania Region (Southern Italy), and that already investigated lentil and grass peas from Valle Agricola [14,15], as well as beans from Gallo Matese [16], getting insight into their biochemical and nutritional properties, chickpea seeds (*Cicer arietinum* L.) from Valle Agricola (hereafter Valle Agricola chickpea) were of interest. Valle Agricola chickpea is classified as a typical product from the Campania Region [17]. This chickpea is morphologically characterized as a small seed, very light hazel colour, thin skin (integument), highly digestible, with a distinguishing and intense flavour, and valuable organoleptic features. It is mainly used dried, for the preparation of traditional dishes of the cultivation area, linked to family and local consumption [17]. Thus, as typical products represent an attractiveness of our agri-food system, nutritional value knowledge of Valle Agricola chickpea is of great interest. In fact, while many studies on the nutritional value of cultivated chickpea seeds are available [18,19], there is virtually no information on those of Valle Agricola chickpea. As it is widely recognized that the strict link with the territorial context provides a production model based on the coevolution, over a very long period of time, of the cultivation/breeding and processing systems of the product within its territory and the resources present in it, getting insights into the nutritional traits of Valle Agricola chickpea could be a springboard for making its beneficial effects broadly known and for better supporting its cultivation and marketing. With this premise, Valle Agricola chickpea, beyond analyses aimed at defining ash, moisture, and macronutrient content, is investigated for its amino acid profile, lipid features, and bioactive compounds. Mineral content, as well as a deep investigation of antinutritional or fiber components, was also pursued, whereas an in vitro protein digestibility assay was applied for acquiring useful data on Valle Agricola chickpea intake.

## 2. Materials and Methods

### 2.1. Chemicals and Reagents

The sources of the chemicals have been described previously [14,16,20]. Particular experimental details are listed below.

### 2.2. Plant Material and Sampling

Valle Agricola chickpea was grown from the in situ reference field in typical soil and illumination conditions in Valle Agricola, ~70 km northwest of Caserta, Italy (geographical coordinates: 41°25′ N 14°15′ E). All the plants were grown during the growing seasons 2017 and 2018, according to a randomized block design with three replicates, each replicate formed by 50 individual plants. Chickpea seeds obtained from randomized block at crop maturity were harvested manually and sun-dried for some weeks. Seeds after harvesting were cleaned to remove broken seeds, dust and other undesirable matter. Dried chickpea seeds were powdered with the Cyclone Sample Mill Instrument (PBI International, Milan, Italy), until flour of a homogeneous size was obtained. The material, hereafter “seed flour”, was stored at a temperature of −80 °C in dark plastic bags.

### 2.3. Ash, Moisture Content and Macronutrient Content

The ash content, moisture level and total crude protein (nitrogen factor of 6.25) were determined according to the AOAC official method [21]. Total lipid (by using Soxhlet apparatus using CHCl_3_ as extracting solvent) and carbohydrate (FAO, 2003) content was determined as previously reported [14,16].

### 2.4. Amino Acid Composition

Free amino acid composition was obtained using seed flour (~200 mg in triplicate) subjected first to ethanol precipitation and then, the pellet was solubilized in sulfosalicylic acid as previously reported [14,22]. Instead, to obtain total (free plus protein) amino acids composition, ∼10 mg of seed flour were hydrolysed for 24 h with 6 N HCl containing 0.02% phenol and then treated as previously reported [14]. Samples with (total amino acids composition) and without hydrolysis (free amino acids composition) were analyzed on a Biochrom 30 amino acid analyzer (Biochrom, Cambridge, UK). As internal standard *nor*-Leu was used.

### 2.5. Determination of Trypsin and Chymotrypsin Inhibitory Activities

In order to determine Trypsin and anti-trypsin activities, as well as chymotrypsin and anti-chymotrypsin activities, the TAME (p-toluenesul-fonyl-L-Arginine methyl ester) and BTEE (N-benzoyl-L-Tyrosine ethyl ester) were used as substrates, respectively. The IC_50_ values of trypsin and α-chymotrypsin activities (i.e., the half maximal inhibitory concentration) of raw soluble chickpeas protein extract were calculated following a procedure already described [14,16]. Raw soluble proteins were extracted from seeds (collected in 2017 and 2018) with and without thermal treatment obtained by boiling chickpea seeds in tap water as previously reported [14,16]. Following the protein extraction, the protein concentration determination by Bio-Rad Protein Assay kit and samples were subjected to spectrophotometric assays.

### 2.6. Digestibility of Proteins In Vitro

An in vitro pepsin-chymotrypsin (P-C) or pepsin-trypsin (P-T) enzyme system was used to determine the resistance of raw soluble chickpeas protein extract to gastro-intestinal (GI) digestion as previously reported [23] with few modifications. 200 μg of raw soluble chickpeas protein extracts with and without thermal treatment at 0, 30, 60 and 120 min (paragraph 2.5) were dissolved in 0.1 M HCl (100 μL). The ratio protease/substrate (E:S) was fixed at 1:100 for digestion conducted at 37 °C for 1 h. Subsequently, pepsin inactivation was obtained by adding 20 μL of 0.1 M NaOH and then mixed with sample buffer (40 μL) in presence of 2-mercapthoethanol. Furthermore, 20 μL (40 μg) of protein extract after 0 min and 60 min of incubation were analyzed by SDS-PAGE. To verify also the effect of chymotrypsin or trypsin, after pepsin hydrolysis, 50 μL of samples were subjected to chymotrypsin or trypsin digestion (E:S = 1:100) after pepsin inactivation by adding 0.1 M NaOH (final volume 100 μL), and incubating for additional 1 h at 37 °C. Aliquots (40 μg) were collected, and hydrolysis was terminated by boiling samples for 10 min, lyophilizing and then mixing with sample buffer (80 μL) before SDS-PAGE in reducing conditions, loading 20 μL (10 μg). SDS-PAGE was carried out as previously described [24].

### 2.7. Determination of Minerals Content

Aliquots of the powdered samples (~250 mg) were mineralized in a Milestone Microwave Laboratory Systems (Ethos 900), endowed with temperature control, by a combination of hydrogen peroxide and nitric acid (H_2_O_2_ 50% *v/v*: HNO_3_ 65% *v/v* = 1:3). After digestion, the solutions were diluted by deionized water to a final volume of 50 mL. The nutrient (Na, Ca, Mn, Fe, Zn, Mg, Cu, K) concentrations were quantified by atomic absorption spectrometry (SpectrAA 20 Varian) via flame furnace using standard solutions (STD Analyticals, Carlo Erba, Sabadell (Barcelona), Spain). Accuracy was checked by analysis of standards (Resource Technology Corporation, Laramie, WY, USA) and the recovery was in a range of 90–110% for each element. All the analyses were performed in triplicate and expressed as mean ± SD.

### 2.8. Chemical Composition Insight by Means of UHPLC-TOF-MS and TOF-MS^2^ Analyses

#### 2.8.1. Sample Preparation

**Figure 1:**
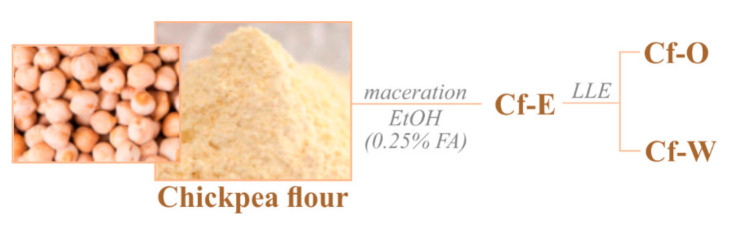
Extraction and fractionation scheme applied to chickpeas before UHPLC-HRMS analysis (FA = formic acid; LLE = liquid/liquid extraction).

Chickpea flour (6 g) underwent maceration, stirring at room temperature for 1 h, using acidified ethanol (0.25% formic acid) as extracting solvent and a matrix/solvent ratio equal to 1:5 (5 mL per g of matrix). Then, the crude extract (Cf-E, Figure 1) was centrifuged at 4500 rpm for 10 min in an Avant^TM^ J-25 centrifuge (Beckman Coulter, Brea, CA, USA), equipped with a JA-14 rotor. The obtained supernatant was dried using a rotary evaporator (Heidolph Hei-VAP Advantage, Schwabach, Germany) and further fractionated by discontinuous liquid/liquid extraction in an *n*-hexane:methanol:water (30:30:8.5) mixture, giving rise to an apolar organic fraction (Cf-O) and an aqueous one (Cf-W).

#### 2.8.2. UHPLC Parameters

Cf-W fraction was analysed with a Shimadzu NEXERA UHPLC system equipped with a Luna Omega Sugar column (3 μm particle size, i.d. 100 mm × 2.1 mm i.d., Phenomenex, Torrance, CA, USA). Chromatographic separation was optimized with an elution gradient of water (A) and acetonitrile (B), both with 0.1% formic acid. The mobile phase composition linearly ramped from 85% B to 65% B in 5 min, where it held for 3 min. Instead, Cf-O elution was achieved on a Luna Omega C18 column (1.6 μm particle size, 150 × 2.1 mm i.d., Phenomenex) using the following gradient conditions: 0–3 min, 20 → 55% B; 3–7 min, 55 → 75% B; 7–10 min, 75 → 95% B; 10–12 min, 95% B. At the end of each run the starting conditions were restored and the column was allowed to re-equilibrate for 2 min. In both cases the flow rate was 0.5 mL min^−1^ and the injection volume was 2.0 μL.

#### 2.8.3. TOF-MS and TOF-MS^2^ Parameters

HRMS analysis was performed using the AB SCIEX TripleTOF 4600 hybrid system (AB Sciex, Concord, ON, Canada) with a DuoSpray ion source operating in negative electrospray ionization. The APCI probe of the source was used for fully automatic mass calibration using the Calibrant Delivery System (CDS).

Data were collected by information dependent acquisition (IDA) using a TOF-MS survey scan of 100–1200 Da (250 ms accumulation time) and eight dependent TOF-MS/MS scans of 80–1000 Da (100 ms accumulation time). Declustering potential (DP) was set at 60 or 75 and collision energy (CE) at 40 or 55. The values were optimized for each class of compounds, with a spread of 15 or 35 V. The following parameter settings were also used: ion spray voltage, −4500 V; ion source heater, 600 °C; curtain gas, 35 psi; ion source gas, 45 psi. Data processing was performed using the PeakView-Analyst TF 1.7 software.

### 2.9. Statistical Analysis

All the analyses were repeated three times and data are expressed as mean ± standard deviation (SD). Data analysis was by Excel Office 2016 (Microsoft Corporation, Redmond, WA, USA). The IC_50_ values were calculated based on inhibition curves: the residual enzyme activities were plotted versus different concentrations of protein extract by fitting data with a nonlinear regression analysis on a semilogarithm scale by using the GraphPad Prism 5 software (GraphPad Software Inc., San Diego, CA, USA). The Bonferroni post-test was used to determine significant differences.The test was performed using a *p* < 0.05.

## 3. Results and Discussion

### 3.1. Nutritional Values

**Figure 2:**
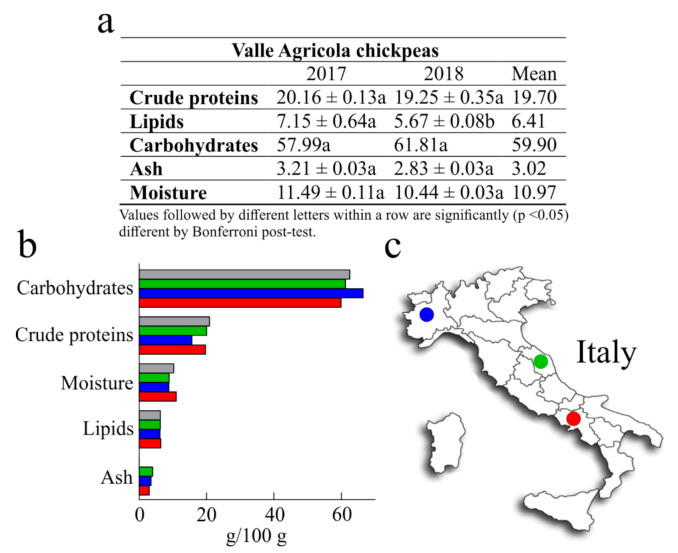
(**a**), nutritional values of Valle Agricola chickpeas collected in the years 2017 and 2018. Values are means (±SD) of triplicate analyses (*n* = 3) and are expressed on dry-weight basis (g/100 g). (**b**), average values of Valle Agricola chickpeas seeds (■) compared with those of common Italian chickpeas reported by the Centro di Ricerca per gli Alimenti e la Nutrizione (■), and with Merella (■) and Alta Valle di Misa (■) Italian chickpeas grown in Piemont and Marche regions. (**c**), geographic localization of Merella (⬤) and Alta Valle di Misa (⬤) chickpeas, grown in Piedmonte and in Marche, two northern and central Italian regions, respectively, while in red (⬤) geographical position of Valle Agricola town (Southern Italy).

The nutritional values of chickpeas have been reported by a number of scientific works [18,25]. Nevertheless, the nutritional value is influenced by genetic and environmental factors, which is why it is important to study the locally grown cultivars in order to assess their nutritional quality. In light of this, the present work was undertaken to determine the nutritional values of Valle Agricola chickpeas, in order to provide useful information for current research in nutrition and food science, and to promote seed sale and consumption. The nutritional values of Valle Agricola chickpea seeds obtained by analysing two samples collected in two different years (2017 and 2018) and their mean are reported in Figure 2. Considering that the two samples have no statistical differences, except for lipid content, we compared the average values with those of common Italian chickpeas reported by the Centro di Ricerca per gli Alimenti e la Nutrizione [26], and with the values of both Merella and Alta Valle di Misa Italian chickpeas grown in the Piemonte and Marche regions [27].

Generally, the crude protein content of different chickpeas represents 12% to 30% of seed with an average value that is commonly 2–3 times higher than that of cereal grains [18]. In this study, the average amount of crude protein from Valle Agricola chickpeas (19.70 g/100 g) was about 6% lower than “CREA” chickpeas (20.90 g/100 g) and about 20% higher than Merella chickpeas (15.70 g/100 g), while similar to the crude protein content of Alta Valle di Misa (2% of difference). Moreover, six landraces collected in Central and Southern Italy as “Cece nero”, a black-seeded chickpea, “Cicerale”, “Guardia dei Lombardi”, “Maglianico”, “Sassano”, and “Spinazzola” show an interval of crude protein content from 19.40 g/100 g to 20.3 g/100 g [28] that includes the crude protein content of Valle Agricola chickpeas.

Raw chickpeas are also lower in lipid content (around 6%) [25]; this value is in accordance with the lipid content retrieved in Valle Agricola chickpeas (6.41 g/100 g). Moreover, lipid content of Valle Agricola chickpeas was higher than that reported for Merella (6.20 g/100 g; ~3%), Alta Valle di Misa and Italian chickpeas (both 6.30 g/100 g; ~2%).

Furthermore, carbohydrate content of Valle Agricola chickpeas represents ~60%; this value is ~ 10%, 2% and 4% lower than Merella, Alta Valle di Misa and Italian chickpeas, respectively. Overall, these data confirm that carbohydrate content in chickpeas represents the major fraction of seed, amounting to about 60% in raw dry chickpeas [18,25].

In addition, as reported in Figure 2, ash and moisture content were determined. In particular, considering that the mean values of ash are about 2% and 4% [18], the ash value in Valle Agricola chickpeas (~3%) was lower than that reported for Merella (3.50 g/100 g; ~14%) and Alta Valle di Misa (4.0 g/100 g; ~24%), while this value is not reported for Italian chickpeas [26]. On the other hand, the average amount of Valle Agricola chickpeas moisture (10.97 g/100 g) was about 20% higher than Merella (8.80 g/100 g) and Alta Valle di Misa (8.90 g/100 g) chickpeas and slightly higher (~6%) with respect to the moisture content of Italian chickpeas.

Finally, some studies regarding the characterization of nutritional traits of several chickpeas originating from Sicily (region of Southern Italy), report that these chickpeas have an average crude protein, lipid and carbohydrate content of 19.5 g/100 g, 6.0 g/100 g and 62.0 g/100 g, respectively. In this case, the crude protein and lipid contents of Sicilian chickpeas are lower with respect to Valle Agricola chickpeas (about 2% and 3%, respectively), while the carbohydrate content is higher (4%) in Sicilian chickpeas [29].

Overall, the consumption of 100 g of dried Valle Agricola chickpeas can provide a caloric intake of about 366 Kcal (~60% carbohydrates, ~20% proteins and ~6% lipids), capable of meeting about 15% of the adult average energy requirement.

### 3.2. Amino Acid Content

Total amino acid content (free plus protein) from hydrolysated Valle Agricola chickpeas was obtained by analyzing the seeds collected in 2017 and 2018, and their mean is reported in Table 1; no statistical differences were retrieved except for arginine (Arg) and glutamic acid plus glutamine (Glx). Despite this, the mean values were compared with those of Italian chickpeas [26], showing qualitative and quantitative differences.

Glx (glutamic acid + glutamine; 3.38 g/100 g; 18%) was the most abundant among the total amino acids in Valle Agricola chickpeas, followed by arginine (Arg; 1.89 g/100 g; 10%), Asx (aspartic acid + asparagine; 1.83 g/100 g; ~10%), lysine (Lys; 1.42 g/100 g; 8%), leucine (Leu; 1.31 g/100 g; 7%), serine (Ser; 1.23 g/100 g; 7%) and phenylalanine (Phe; 1.23 g/100 g; 7%), which represented about 66% of the total amino acids. In addition, the amount of essential amino acids (His, Ile, Leu, Lys, Met, Phe, Thr, Val (Trp; tryptophan is not included as it was not determined in the total hydrolysed samples) see Table 1) in Valle Agricola chickpeas was 7.12 g/100 g (~40% of total). The amount of methionine plus cysteine after also performic acid treatment in Valle Agricola chickpeas was 0.57 g/100 g (~3% of total), confirming the low level of sulphur amino acids as reported for other legumes. However, the sulphur amino acids content in Valle Agricola chickpeas is slightly higher than that reported for Italian chickpeas (0.48 g/100 g; 2.4% of total). On the other hand, Italian chickpeas contained high levels of amino acids Glx (3.41 g/100 g; 17%), Asx (2.40 g/100 g; 12%), Arg (1.92 g/100 g; ~10%), Leu (1.61 g/100 g; 8%), Lys (1.43 g/100 g; 7%) and Phe (1.27 g/100 g; 6%), which show a different succession with respect to Valle Agricola chickpeas, but similar percentages. Furthermore, histidine (His), threonine (Thr), alanine (Ala) and serine (Ser) were more abundant in Valle Agricola chickpeas than in Italian chickpeas. Finally, significant statistical differences between seeds collected in 2017 and 2018, found for arginine and glutamic acid plus glutamine (Glx), are likely associated with nitrogen availability [30].

**Figure 3:**
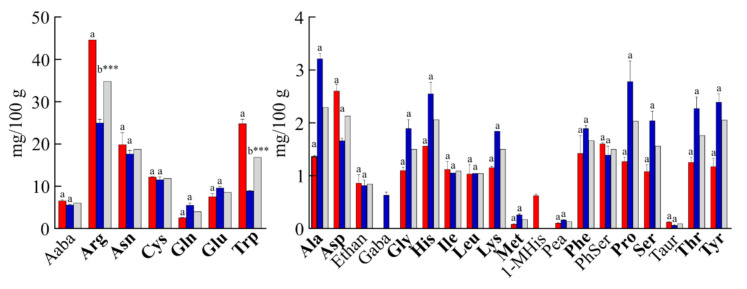
Free amino acid composition of Valle Agricola chickpeas. Values are means (± SD) of triplicate analyses (*n* = 3) and are expressed on dry-weight basis (mg/100 g). Valle Agricola chickpea seeds were collected in the years 2017 (red) and 2018 (blue), in grey the average values. Protein amino acids are highlighted in bold. Code for non-protein amino acids: Aaba, α-Aminobutyric acid; Ethan, ethanolamine; GABA, γ-aminobutyric acid; 1-MHis, 1-methyl-L-histidine; Pea, phosphorylethanolamine; PhSer, phosphoserine; Taur, L-taurine. Different letters (a, b) indicate a significance difference among levels of free amino acids (*p* < 0.05) by Bonferroni post-test. *** indicates significant difference at *p* < 0.001.

In terms of free amino acids, the average total amount in Valle Agricola chickpeas was ~129 mg/100 g of dry-weight (Figure 3). Asparagine (Asn) was by far the most abundant among free protein amino acids (~15% of total), not considering Arg and tryptophan (Trp), which differ between the two samples analysed. Furthermore, cysteine (11.85 mg/100 g), glutamic acid (8.57 mg/100 g) and valine (4.51 mg/100 g) were the most abundant free amino acids in Valle Agricola chickpeas, while the amount of each of the other protein amino acids did not exceed 24.93 mg/100 g of product (19% of total free amino acids content). The analysis also evidenced the presence of five nonprotein amino acids (i.e., α-Aminobutyric acid (Aaba); ethanolamine (Ethan); phosphorylethanolamine (Pea); phosphoserine (PhSer) and L-taurine (Taur)) present in both collected years, and the amount was 8.61 mg/100 g (~7% of total). Moreover, 1-methyl-L-histidine (1-MHis) and γ-aminobutyric acid (GABA) were detected only in 2017 or 2018, respectively. The free amino acid content of other chickpeas seeds has not been published previously.

However, significant statistical differences between seeds collected in 2017 and 2018 were found for arginine and tryptophan in free amino acid composition. The high amount of free arginine was already reported in chickpeas [31], and this different content could be associated with nitrogen availability [30], while the distinct tryptophan content could be associated with an endogenous inhibitor of embryo germination [32], since it is also been reported in fair quantities in chickpeas seeds [33].

### 3.3. Anti-Proteinase Inhibitor Activity

**Figure 4:**
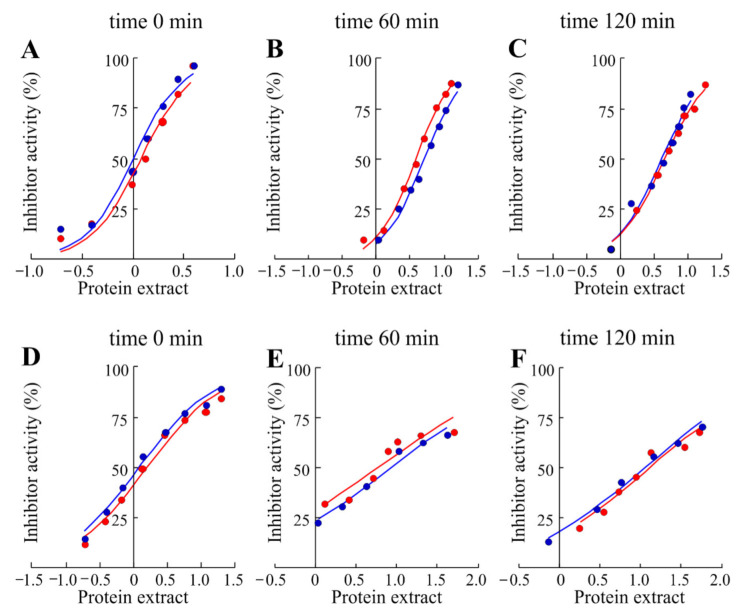
Inhibition curves of trypsin and α-chymotrypsin by raw soluble protein extract obtained from Valle Agricola chickpeas (**A**, **D**, respectively) or cooked Valle Agricola chickpeas at 60 (**B**, **E**, respectively) and 120 min (**C**, **F**, respectively). Increasing concentrations of raw protein extract were added to a fixed concentration of enzymes as indicated in Material and Methods. (⬤) and (⬤), seeds collected in 2017 and 2018, respectively.

Plant seeds used as nutritional food contain many compounds that are classified as non-nutritive and known as ANCs (antinutritional compounds). These non-nutritive compounds are different and belong to classes of proteins (enzymes, lectins or protein inhibitors) or to other smaller molecules including phytates, saponins, alkaloids, tannins and glycosides complex that reduce nutrient utilization or food intake [34]. In particular, protein intake process is limited by the presence of protease inhibitors that inhibit proteolytic enzymes such as trypsin and chymotrypsin [35]. In this framework, the cooking [36] or germinating [37] seed processes inactivate or decrease the content of these proteolytic inhibitors, improving protein digestibility and increasing amino acid availability. In this contest, we have tested antitrypsin and anti-chymotrypsin activities of raw soluble chickpeas protein extract from Valle Agricola chickpeas with and without the boiling process. The inhibitory activities obtained from the protein extracts (Figure 4) were reported as average IC_50_ values per year.

Antitrypsin activity of Valle Agricola chickpeas without thermal treatment (IC_50_ 1.10 μg of raw soluble protein extract) was ~21% higher than the anti-chymotrypsin activity (IC_50_ 1.40 μg of raw soluble protein extract), while no protease activities were detected in the same experimental conditions. On the other hand, to confirm the decreasing effect of the boiling process on trypsin inhibitor content, aliquots of Valle Agricola seeds were boiled for 60 and 120 min, and their anti-protease activities were re-assayed. After a thermal treatment of 60 min, antitrypsin and anti-chymotrypsin activities were respectively ~75% (IC_50_ 4.45 μg of raw soluble protein extract) and ~82% (IC_50_ 7.08 μg of raw soluble protein extract) lower than those detected in raw soluble proteins from chickpea seeds. On the other hand, antitrypsin and anti-chymotrypsin activities after 120 min were respectively ~74% (IC_50_ 4.30 μg of raw protein extract) and ~88% (IC_50_ 12.10 μg of raw soluble protein extract) lower than those detected in raw chickpea seeds. In addition, the thermal treatment (boiling process of chickpea seeds) shows the decrease in raw soluble proteins recovered from chickpeas seed extract as average value, from 19.25 mg/mL to 1.17 mg/mL and 0.80 mg/mL at 60 and 120 min, respectively, see Table S1. Moreover, as previously reported for other legumes, the boiling process reduces antiprotease activities of Valle Agricola chickpea seeds [36]. In particular, the thermal treatment has a higher effect on anti-chymotrypsin with respect to antitrypsin activities in these seeds.

### 3.4. Digestibility of Soluble Raw Chickpeas Proteins Extract In Vitro

In order to verify the digestibility of raw soluble chickpeas protein extracts with and without thermal treatment despite the presence of residual antitrypsin and anti-chymotrypsin activities, an in vitro study on digestibility of these extracts was carried out. Therefore, we treated these extracts with the common digestive pepsin-chymotrypsin or pepsin-trypsin system [23] at different times, and the effect was evaluated by SDS-PAGE.

**Figure 5:**
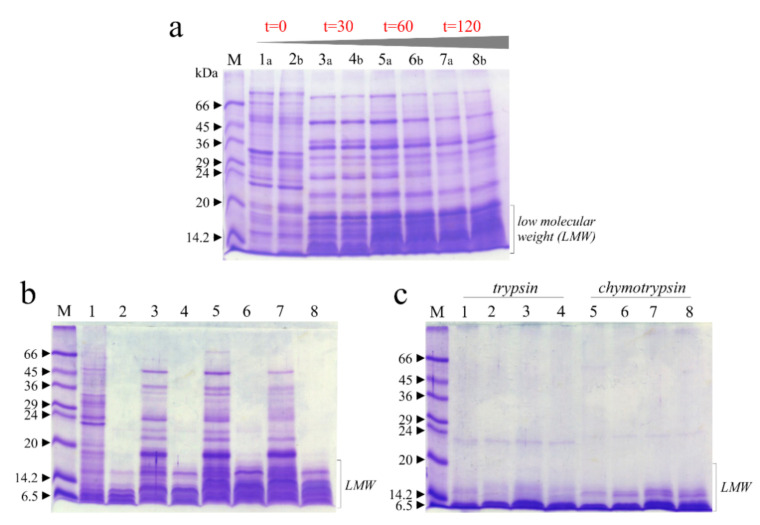
Raw soluble protein extracts from chickpea seeds subjected to in vitro protein digestibility with and without thermal treatment (boiling). (**a**), SDS-PAGE profiles of extracts collected in 2017 (**a**) and 2018 (**b**). Lanes 1 and 2, 3 and 4, 5 and 6, 7 and 8, extracts after thermal treatment (0, 30, 60 and 120 min, respectively). (**b**), SDS-PAGE profile of mixture extracts from seeds collected in 2017/2018 subjected to pepsin treatment. Lanes 1, 3, 5 and 7 boiled samples for 0, 30, 60 and 120 min without subsequent pepsin treatment. Lanes 2, 4, 6 and 8, boiled samples for 0, 30, 60 and 120 min with subsequent pepsin treatment (60 min). (**c**), SDS-PAGE profile of extracts subjected to trypsin or chymotrypsin treatment. Lanes 1, 2, 3, and 4, extracts after boiling (0, 30, 60 and 120 min; respectively), pepsin pretreatment (60 min) and subsequent trypsin treatment (60 min). Lanes 5, 6, 7 and 8, extracts after boiling (0, 30, 60 and 120 min; respectively), pepsin pretreatment (60 min) and subsequent chymotrypsin treatment (60 min). M, molecular weight markers. SDS-PAGE was carried out in 15% polyacrylamide separating gel under reducing conditions.

In this framework, in Figure 5a is reported as reference the SDS-PAGE profile of raw soluble chickpea protein extracts with and without thermal treatment boiling samples at 0, 30, 60 and 120 min, in which it is evident that the thermal treatment changed the profile of protein bands and increased the quantity of low molecular weight (LMW) bands. Subsequently, pepsin digestion displays that most of the extract is hydrolyzed in acid conditions (Figure 5b), with residual LMW bands, present in higher amount when samples were treated for longer times (Figure 5b). The same LMW bands disappeared when samples were pretreated with pepsin and then subjected to chymotrypsin or trypsin digestion (Figure 5c).

Overall, the residual antitrypsin and anti-chymotrypsin activities do not interfere with the digestion of raw soluble chickpea proteins; in fact, the boiling process favorably depleted these precious seeds of intrinsic antinutritional factors.

### 3.5. Mineral Content

The different mineral content present in chickpea seeds is displayed in Table 2. Raw chickpea seeds (100 g) as mean between two years assayed, provide for about 2.4 mg/100 g of Na, 200 mg/100 g of Ca, 0.45 mg/100 g of Mn, 4.32 mg/100 g of Fe, 4.2 mg/100 g of Zn, 164 mg/100 g of Mg, 0.83 mg/100 of Cu and 748 mg/100 g of K.

Chickpea seeds showed very good levels of several mineral nutrients, especially magnesium, potassium, calcium, zinc and manganese, data comparable to that reported by the food composition database for CREA [26]. As it is well known, the correct nutrient supply is important for human health. For example, P is indispensable in metabolism and in the stimulation of muscle contractions; K is an important element in the functioning of the skeletal muscles and the myocardium and in the regulation of excitability neuromuscular; Fe is an indispensable element in the processes of cellular respiration as well as in collagen synthesis and in the metabolism of nucleic acids; Ca is an important element in the regulation of muscle contraction and in the construction of the skeleton and teeth; Na is an important regulator of cell membrane permeability [38]. By comparing two sampling years, lower mineral content was observed in chickpea seeds sampled in 2017 than in 2018. These differences were significant (almost for *p* < 0.05) among all minerals assayed except for Na and Cu, even if there was a trend. This might be due to low rainfall that preceded the month of sampling for chickpeas collected in 2017 (30–40 mm of rain) than in 2018 (70–80 mm of rain) as reported by Alife weather station (latitude 41.329 N, longitude 14.33 E [39]). As known, in fact, there is a reduction in absorption by the whole plant after the drought period [40,41,42].

### 3.6. UHPLC-HRMS Profiling of Cf-W Extract

In order to unravel their chemical composition, Cf-W extracts of chickpeas from both collection years were chemically characterized by means of UHPLC-TOF-MS and TOF-MS^2^ techniques and their profiles appeared almost superimposable. HR-MS and HR-MS/MS data, acquired in ESI negative ion mode, were summarized in Table 3. Hypothesizing to deal mostly with carbohydrates, for chromatographic separation a HILIC stationary phase was chosen, in order to improve polar compound retention and selectivity. Indeed, if the selectivity was guaranteed for sugar compounds, this was not true for glycerophospholipids, and any attempt to reach a greater peak resolution failed. However, based on different m/z values of the precursor ions, five compounds (**1–5**) belonging to this class were recognized and tentatively identified. They are constituted by a glycerol backbone, which is alkylated or acylated at sn-1 and/or sn-2 position and a phosphate group at sn-3 position. Esterification to this latter with different polar head groups gives rise to various subclasses. Within each class, the chemical diversity is due to the presence of a number of fatty acid residues [43].

**Figure 6:**
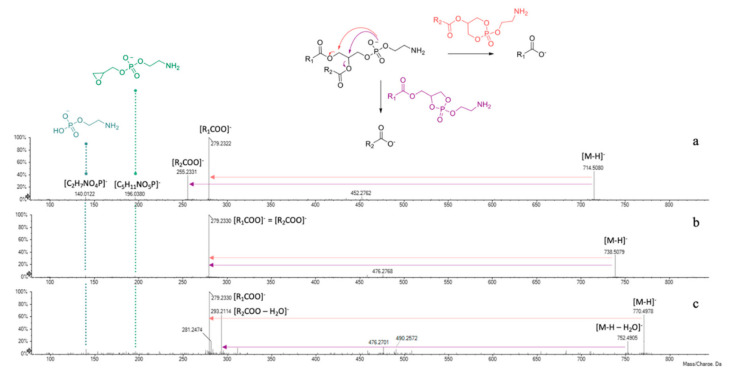
ESI-TOF/MS^2^ spectra of compounds (**a**) **1**, (**b**) **2** and (**c**) **3** and fragmentation scheme of GPE derivatives.

Taking into account literature data, compounds **1**–**3** were tentatively identified as glycerophosphatidylethanolamine (PE) derivatives (Table 3). In fact, negative mode ESI-MS/MS spectra showed two characteristic product ions at m/z 140.0122 and 196.0380, corresponding to 2-aminoethyl hydrogen phosphate ([C_2_H_7_NO_4_P]^−^) and 2-aminoethyl (oxiran-2-ylmethyl) phosphate ([C_5_H_11_NO_5_P]^−^) moieties, respectively (Figure 6).

The presence of fragments at m/z 279.2322(30) and 255.2331 gave information about the fatty acids linked to the glycerol backbone of compound 1, being indicative of 18:2 (e.g., linoleic acid) and 16:0 (palmitic acid) residues. The lower relative intensity of this latter suggested its sn-2 linkage. In fact, the hypothesized fragmentation mechanism, depicted in Figure 6, involves the attack of the negatively charged phosphate group at C-1 or C-2 glycerol carbon atoms, leading to the formation of five- or six-membered ring structures. It is likely to assume that at high collision energies (40–50 eV) the preferred fragmentation pathway is the one giving rise to sn-1 carboxylate anion [44]. Compound 2 was tentatively identified as GPE (18:2/18:2). Indeed, the tandem mass spectrum showed only one main product ion (m/z 279.2330), allowing us to hypothesize the presence of the same unsaturated fatty acid residue (e.g., linoleic acid) both at sn-1 and sn-2 positions. A further confirmation of the occurrence of a 18:2 fatty acid in GPEs 1 and 2 can be found in the less intense fragments at m/z 452.2762 and 476.2768, respectively, likely due to the neutral loss of a C_18_H_30_O residue [45]. 

The 18:2 fatty acid chain belongs also to compound 3, appearing as base peak in its TOF-MS^2^ spectrum (Figure 6c). Moreover, the deprotonated molecular ion underwent collision-induced dissociation, losing a water molecule (m/z 770.4968 → 752.4905) and then gave rise to the ion at m/z 293.2114 through the five-membered ring formation mechanism. Based on experimental data, this latter was hypothesized to be a dehydrated dihydroxyoctadecadienoic acid (e.g., diOH-linoleic acid), linked to glycerol at sn-2 position.

In negative ion mode mass spectrometry experiments, they were previously described as adducts with the counter ion [43], which in the present discussion is represented by formate anion. The ion at m/z 168.0450(1), corresponding to a 2-(dimethylamino)ethyl hydrogen phosphate structure ([C_4_H_11_NO_4_P]^−^), was pivotal to further confirm the polar head group of phosphatidylcholine. The fragmentation pathway of these molecules provided at first the loss of the counter ion and of an additional *N*-methyl group, giving rise to the corresponding dimethyl-phosphatidylethanolamine derivatives (at m/z 742.5443 and 766.5360 for compounds 4 and 5, respectively).

**Figure 7:**
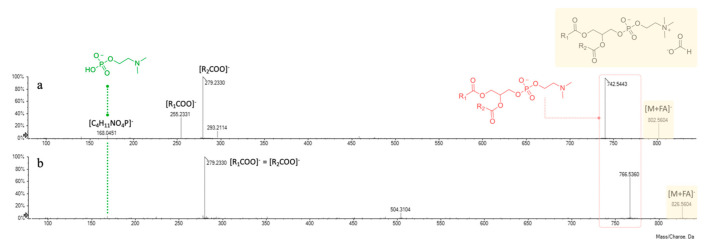
ESI-TOF/MS^2^ spectra of compounds 4 (**a**) and 5 (**b**).

Then, the product ions of unsaturated 18:2 and 16:0 fatty acid residues occurred at m/z 255.2331 and 279.2330, likely formed through the mechanism detailed above for GPEs. As previously reported, GPCs prefer to fragment by the formation of five-membered rings [44], thus providing as the most intense peak the [R_2_COO]^−^ ion (Figure 7a). It has been previously reported that polar lipids, among which phospholipids such as phosphatidylethanolamine and phosphatidylcholine derivatives, are the most abundant compounds of defatted chickpea flour and a possible explanation of this evidence relies in the awareness that they are constituents of cell organelles’ membrane (e.g., endoplasmic reticulum or Golgi apparatus) [46].

**Figure 8:**
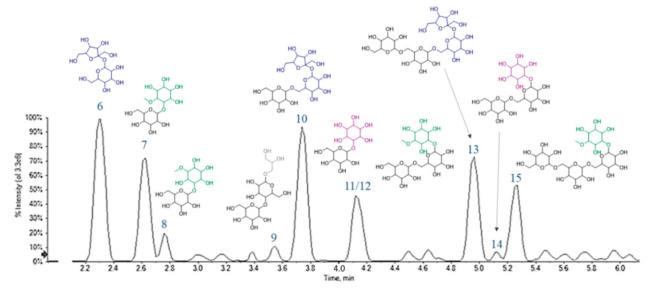
A representative chromatogram of Cf-W fraction, in which the tentatively assigned chemical structures of sugar derivatives are assigned to each peak.

Compounds 6–15 were tentatively identified as carbohydrate derivatives. Their elution order on the Luna Omega Sugar column was influenced by the number of saccharidic units, in that a higher number of units was directly translated in a greater retention time. Moreover, on an equal number of units, the presence of a cyclitol moiety implied a delay in the elution (Figure 8).

Based on HR-MS experiments, a molecular formula C_12_H_22_O_11_ was assigned to two compounds (6 and 11), that at least in principle could be associated with the presence of di-hexoses. However, even if TOF-MS^2^ spectra were almost superimposable, the ion at m/z 125.0242 (Table 3) of compound 11 made the difference. It could correspond to a benzene-1,3,5-triol structure (phloroglucinol; C_6_H_6_O_3_), deriving from three dehydration steps of a cyclitol moiety (m/z 179.0566). Thus, they were tentatively identified as dihexose (e.g., sucrose, 6) and galactinol (11). Metabolites 7 and 8 (C_13_H_24_O_11_) appeared to be methyl-derivatives of this latter, hence formed by a pinitol unit (m/z 193.0708(19); Table 3) and a hexose (e.g., galactose). The other fragments, such as those at m/z 179.0566, 161.0448, 119.0347 and 89.0245, were those typical of disaccharides, well known in literature [20]. Based on TOF-MS and TOF-MS^2^ data, compound 9 was putatively identified as di-galactosylglycerol, a soluble carbohydrate previously extracted from the seeds of some legume species [47]. 

Compounds 10 and 13 are a tri- and tetrasaccharide, likely belonging to the so-called raffinose-series oligosaccharides (RFOs) [14], whereas metabolite 14 could be the reduced form of 10, bearing a cyclitol unit instead of a hexose. RFOs and the other α-galactosides are important in plant physiological processes, as seed germination [48] and seed desiccation and stress tolerance [49]. When highly assumed with the diet, they are considered antinutritional factors for humans who, lacking α-galactosidases, are not able to digest them. Hence, some embarrassments, such as flatulence, occur, and only soaking chickpeas overnight before cooking could reduce these phenomena, decreasing RFO levels [50].

The deprotonated ion at m/z 517.1773 (C_19_H_34_O_16_) with an RDB value of 3 for compound 12 was in accordance with ciceritol, a pinitol digalactoside very commonly found in chickpeas, like the other α-galactooligosaccharides (e.g., 15, a galactosylciceritol). It has been demonstrated that ciceritol exerts a prebiotic effect, by improving the human colon microflora and enhancing the production of short chain fatty acids with positive implications for human health [51].

Very few other compounds were detected, among which were three isoflavones and two hydroxybenzoic acid derivatives. These latter ones have been putatively characterized as hydroxybenzoic acid hexosylpentoside (19; m/z 431.1204) and dihydroxybenzoic acid hexoside (20; m/z 315.0721). In both cases the loss of sugar moiety, as 294 (162 + 132) Da or simply 162/163 Da, generated aglycone ions at m/z 137.0244 (C_7_H_5_O_3_^−^) and 153.0185/152.0111 (C_7_H_5_O_4_^−^/ C_7_H_4_O_4_•^−^), respectively.

Finally, according to their molecular formulas (C_16_H_12_O_5_) and TOF-MS^2^ spectra, compounds 16 and 18 were tentatively identified as *O*-methylgenistein isomers, which differed in the position of the methoxy group. Indeed, besides the loss of a methyl radical, cross-ring fragmentations likely occurred via retro-Diels Alder reactions, as previously suggested [52]. A similar hypothesis could be formulated for compound 17 (m/z 267.0664, C_16_H_12_O_4_), which could be a methyldaidzein, for example, formononetin, the major isoflavone found in chickpeas together with biochanin A [53]. Isoflavones have been widely found in the Fabaceae family, including soybean, pea, fava bean, chickpea and lentil [54]. A number of health-promoting functions have been attributed to them [55], among which the most investigated is their capacity to act as phytoestrogens, useful in the treatment of menopausal symptoms and osteoporosis caused by estrogen deficiency [56]. Moreover, methoxylated isoflavones, such as biochanin A and formononetin, could be *O*-demethylated to genistein and daidzein by human intestinal microflora and liver, resulting in an enhancement of their biological properties [57,58].

### 3.7. UHPLC-HRMS Profiling of Cf-O Extract

**Figure 9:**
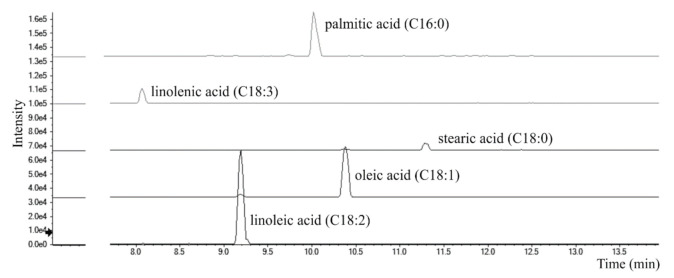
Extracted Ion Chromatograms (XICs) of fatty acids tentatively identified in Cf-O fraction.

Total Ion Chromatograms (TICs) of Cf-O fractions showed very poor signals, all referring to saturated or unsaturated free fatty acids. Evaluating area under peaks, hypothesizing similar ionization efficiency within the same class, the most abundant is linoleic acid (C18:2; m/z 279.2330), followed by oleic (C18:1; m/z 281.2486) and palmitic (C16:0; m/z 255.2330) acids. Traces of linolenic (C18:3; m/z 277.2173) and stearic (C18:0; m/z 283.2643) acids were also detected (Figure 9). Data obtained are in line with those previously reported in literature [59,60,61].

## 4. Conclusions

Presently, the extraordinary recovery of typical agricultural products arouses even greater attention, and biochemistry investigation of characteristic products could be a strategy to favor rural development and multifunctionality in agriculture. 

In this perspective, the endless resource of local and characteristic products of Valle Agricola, a village of ancient origins in the Campania Region, has been taken into account, and the nutritional traits of its tasty chickpea have been analyzed in a short supply chain scenario. In fact, in order to also stimulate economic growth in this territory, and develop social ties at the local level, prerequisite requirements need to be outlined. Thus, a plethora of biochemical approaches was adopted to get insight into the nutrient composition and the metabolic features traits of Valle Agricola chickpeas. This autochthonous legume is a good source of macronutrients, in particular crude proteins (19.70 g/100 g), with a low lipid content, and a valuable content of essential and nonessential amino acids, of which poor sulphur amino acids (methionine and cysteine), as already known for legumes, are poorly present. Furthermore, Valle Agricola chickpeas show a good level of magnesium, potassium, calcium, zinc and manganese, which play fundamental roles in different biochemical and physiological processes in humans. Moreover, glycerophospholipids, free unsaturated fatty acids, together with isoflavone compounds, define the nutraceutical heritage of this landrace, whereas the richness in oligosaccharides makes Valle Agricola chickpeas a good prebiotic source. The intrinsic antinutritional compounds with antitrypsin and anti-chymotrypsin activities, which interfere with protein assimilation, were quantitatively evaluated, with and without thermal treatment (boiling process of seeds). Data acquired show that the boiling treatment reduces the presence of antinutritional compounds even if the retrieved residual heat-resistant antitrypsin and anti-chymotrypsin activities are suggested as a prerequisite of the anticancer properties of chickpeas and in general of legumes. In particular, it is observed that legume consumption is associated with a decrease in the incidence of colorectal cancer [62].

Valle Agricola chickpeas are shown to have a precious reservoir of nutrients and bioactive compounds and have nothing to envy in more well-known chickpeas on the market. This investigation aims at opening up Valle Agricola chickpeas to a new value chain that takes into account the current interest in local and traditional food and the possibility of new opportunities for this niche market.

## Figures and Tables

**Table 1:** Total amino acid composition of Valle Agricola chickpeas compared with Italian database chickpeas (CREA, 2009). Valle Agricola chickpeas seeds were collected in the years 2017 and 2018. Values are means (± SD) of triplicate analyses (*n* = 3) and are expressed on dry-weight basis (g/100 g).

Valle Agricola Chickpeas				CREA Chickpeas
Amino Acid	2017	2018	Mean	
Essential amino acids				
His	0.67 ± 0.05 a	0.55 ± 0.06 a	0.61	0.53
Ile	0.76 ± 0.02 a	0.67 ± 0.01 a	0.71	0.89
Leu	1.42 ± 0.01 a	1.20 ± 0.10 a	1.31	1.61
Lys	1.50 ± 0.02 a	1.35 ± 0.17 a	1.42	1.43
Met	0.24 ± 0.01 a	0.21 ± 0.02 a	0.23	0.23
Phe	1.33 ± 0.01 a	1.14 ± 0.03 a	1.23	1.27
Thr	0.92 ± 0.01 a	0.82 ± 0.02 a	0.87	0.79
Trp	n.d.	n.d.	n.d.	0.22
Val	0.80 ± 0.01 a	0.68 ± 0.03 a	0.74	0.97
Non-essential amino acids				
Ala	0.97 ± 0.00 a	0.79 ± 0.04 a	0.88	0.86
Arg	2.21 ± 0.03 a	1.58 ± 0.07 b *	1.89	1.92
Asx	2.02 ± 0.08 a	1.65 ± 0.05 a	1.83	2.40
Cys ^§^	0.33 ± 0.01 a	0.35 ± 0.03 a	0.34	0.25
Glx	3.76 ± 0.17 a	3.00 ± 0.07 b **	3.38	3.41
Gly	0.83 ± 0.03 a	0.68 ± 0.03 a	0.76	0.81
Pro	0.81 ± 0.07 a	0.57 ± 0.08 a	0.69	0.83
Ser	1.35 ± 0.07 a	1.12 ± 0.01 a	1.23	1.06
Tyr	0.62 ± 0.04 a	0.53 ± 0.02 a	0.58	0.66
**Total**	20.55	16.87	18.71	20.14

For protein amino acids, a three-letter code has been used. Values followed by different letters (a, b) within a row are significantly (*p* < 0.05) different by Bonferroni post-test. *, ** indicate significant at *p* < 0.05 and *p* < 0.01, respectively. ^§^, Cys amount was evaluated after performic acid oxidation. n.d. not determined.

**Table 2:** Mineral element content (expressed on dry-weight basis (mg/100 g)) in the chickpea seeds, sampled during two years (2017 and 2018). Data are mean ± SD. *, significant differences (*p* < 0.05), see main text.

Element	2017	2018	Mean
Na	2.29 ± 0.115	2.52 ±0.126	2.40
Ca	174.6 ± 8.733 *	226.4 ± 11.32 *	200
Mn	0.231 ± 0.011 *	0.668 ± 0.033 *	0.45
Fe	2.025 ± 0.101 *	6.625 ± 0.331 *	4.30
Zn	2.437 ± 0.122 *	5.937 ± 0.297 *	4.20
Mg	141.2 ± 7.058 *	187.7 ± 9.384 *	164
Cu	0.79 ± 0.039	0.87 ± 0.043	0.83
K	645 ± 32.25 *	852 ± 42.6*	748

**Table 3:** TOF-MS and MS^2^ data of compounds tentatively identified in Cf-W extract (RDB = Ring and Double Bond; FA = Formate Anion; GPE = glycerophosphoethanolamine; GPC = glycerophosphocholine). Base peaks in MS/MS spectra are reported in bold.

Peak n.	RT (min)	Tentative Identification	Formula	(M-H)^−^ calc. (*m/z*)	(M-H)^−^ Found (*m/z*)	RDB	Error (ppm)	MS/MS Fragment Ions (*m/z*)
Glycerophoshpolipids								
1	0.603	GPE(18:2/16:0)	C_39_H_74_NO_8_P	714.5079	714.5077	4	−0.3	714.5079; 452.2753; 434.2707; **279.2330**; 255.2330, 196.0363; 140.0125
2	0.604	GPE(18:2/18:2)	C_41_H_74_NO_8_P	738.5079	738.5062	6	−2.3	738.5079; 476.2783; 458.2659; **279.2330**; 196.0363; 140.0113
3	0.608	GPE(18:2/18:2-diOH)	C_41_H_74_NO_10_P	770.4978	770.4961	6	−2.2	770.4978; 752.4895; 293.2122; 281.2500; **279.2330**; 196.0401; 140.0133
4	0.609	GPC(18:2/16:0)	C_43_H_82_NO_10_P	802.5604 (M + FA)^−^	802.5585	4	−2.3	802.5604; 742.5443; 480.3130; 293.2130; **279.2330**; 255.2341; 168.0451
5	0.611	GPC(18:2/18:2)	C_45_H_82_NO_10_P	826.5604 (M + FA)^−^	826.5579	6	−3.0	826.5604; 766.5409; 504.3070; 486.2995; **279.2330**; 168.0436
Carbohydrates								
6	2.237	Dihexose (e.g., sucrose)	C_12_H_22_O_11_	341.1089	341.1076	2	−3.9	341.1089; 179.0557; 161.0456; 149.0455; 143.0352; 131.0347; 119.0352; 113.0245; 101.0247; **89.0248**
7	2.630	Galactopinitol (or methylgalactinol) 1	C_13_H_24_O_11_	355.1246	355.1229	2	−4.8	355.1244; 193.0708; 179.0566; 175.0608; 161.0448; 149.0445; 143.0337; 131.0342; 125.0240; 119.0347; 113.0242; 101.0242; **89.0245**
8	2.781	Galactopinitol (or methylgalactinol) 2	C_13_H_24_O_11_	355.1246	355.1252	2	1.7	355.1250; 193.0719; 161.0458; 157.0509; 141.0197; 125.0255; 119.0355; 113.0254; **101.0253**; 99.0099; 89.0250
9	3.549	Dihexosylglycerol	C_15_H_28_O_13_	415.1457	415.1441	2	−3.9	415.1457; 305.0889; 287.0756; 263.0768; 253.0934; 235.0826; 221.0663; 185.0456; 179.0561; 161.0457; 149.0468; 143.0350; 131.0349; 125.0244; 119.0350; 113.0246; 101.0245; **89.0247**
10	3.740	Trihexose (e.g., raffinose)	C_18_H_32_O_16_	503.1618	503.1614	3	−0.7	503.1627; 341.1077; 323.0983; 281.0877; 251.0769; **221.0669**; 179.0563; 161.0453; 149.0452; 143.0350; 131.0347; 119.0351; 113.0245; 101.0244; **89.0248**
11	3.991	Galactinol	C_12_H_22_O_11_	341.1089	341.1073	2	−0.1	341.1087; 179.0566; 161.0455; 149.0450; 143.0357; 131.0349; 125.0242; 119.0347; 113.0243; 107.0335; 101.0243; **89.0243**
12	4.122	Ciceritol	C_19_H_34_O_16_	517.1774	517.1773	3	−0.2	517.1774; 337.1128; 281.0869; 263.0760; 221.0661; 193.0713; 179.0561; 161.0454; 149.0455; 143.0349; 131.0349; 125.0241; 119.0350; 113.0245; 101.0246; **89.0249**
13	4.967	Tetrasaccharide (e.g., stachyose)	C_24_H_42_O_21_	665.2150	665.2146	4	0.6	665.2152; 503.1611; 485.1504; 443.1403; 425.1305; **383.1194**; 341.1086; 281.0872; 251.0763; 221.0665; 203.0562; 179.0562; 161.0441; 143.0344; 119.0348; 113.0249; 101.0247; 89.0248.
14	5.141	Fagopyritol B2	C_18_H_32_O_16_	503.1618	503.1609	3	−1.7	503.1613; 341.1095; **323.0972**; 281.0898; 263.0760; 221.0651; **179.0560**; 161.0455; 149.0441; 143.0352; 131.0351; 125.0246; 119.0349; 113.0245; 101.0245; 89.0247.
15	5.254	Galactosyl-ciceritol	C_25_H_44_O_21_	679.2302	679.2298	4	−0.6	679.2309; 661.2172; 499.1667; 443.1388; 383.1191; 341.1076; 281.0866; 251.0764; 221.0668; **179.0565**; 161.0456; 143.0353; 131.0354; 125.0249; 119.0351; 113.0248; 101.0249; 89.0251.
*Hydroxybenzoic acids and isoflavones*								
16	0.508	*O*-Methylgenistein 1	C16H12O5	283.0612	283.0608	11	−1.4	283.0612; **268.0385**; 267.0312; 239.0323; 211.0374; 195.0423; 167.0466; 132.0168
17	0.533	*O*-Methyldaidzein	C16H12O4	267.0663	267.0664	11	0.4	267.0663; **252.0419**; 251.0334; 224.0452; 223.0379; 195.0418; 167.0460; 145.0040; 132.0170; 91.0132
18	0.534	*O*-Methylgenistein 2	C16H12O5	283.0612	283.0611	11	−0.3	283.0612; **268.0381**; 267.0311; 239.0331; 211.0373; 195.0412; 167.0469; 132.0164
19	2.315	Hydroxybenzoic acid hexosylpentoside	C18H24O12	431.1195	431.1204	7	2.1	431.1195; 299.0780; **137.0244**; 93.0347; 89.0245
20	5.209	Dihydroxybenzoic acid hexoside	C13H16O9	315.0722	315.0721	6	−0.2	315.0721; 153.0185; 152.0111; 109.0291; **108.0212**

## Data Availability

Data available on request.

## Supplementary Materials

The following are available online at https://www.mdpi.com/2304-8158/10/3/583/s1, Table S1: Soluble raw proteins and IC_50_ of anti-protease inhibitor activity after thermal treatment of chickpeas seeds collected in the years 2017 and 2018. Values are means (±SD) of triplicate analyses (*n* = 3).
