# Supplementary material for: Valle Agricola Chickpeas: Nutritional Profile and Metabolomics Traits of a Typical Landrace Legume from Southern Italy
Source: Foods. 2021 Mar 10;10(3):583. doi: 10.3390/foods10030583 (PMC8002183; doi:10.3390/foods10030583)
Supplement: Supplementary file 1 [file foods-10-00583-s001.pdf]

Table S1. Soluble raw proteins and IC_{50} of anti-protease inhibitor activity after thermal treatment of chickpeas seeds collected in the years 2017 and 2018. Values are means (±SD) of triplicate analyses (n = 3).

Anti-proteinase inhibitor activity	thermal treatment time (min)	raw protein (mg/mL)		IC_{50} (µg proteinase inhibitor)		Mean	
		harvest year					
		2017	2018	2017	2018	raw protein (mg/mL)	IC_{50} (µg proteinase inhibitor)
anti-trypsin activity							
	0	19.11	19.38	1.19	1.01	19.25±0.19	1.10±0.12
	60	1.27	1.06	3.82	5.07	1.17±0.15	4.45±0.89
	120	0.87	0.72	4.48	4.12	0.80±0.11	4.30±0.25
anti-chymotrypsin activity							
	0	19.11	19.38	1.58	1.22	19.25±0.19	1.40±0.25
	60	1.27	1.06	5.81	8.36	1.17±0.15	7.08±1.80
	120	0.87	0.72	12.81	11.39	0.80±0.11	12.10±1.00
